# Folic Acid Supplementation Promotes Mammary Tumor Progression in a Rat Model

**DOI:** 10.1371/journal.pone.0084635

**Published:** 2014-01-21

**Authors:** Shaidah Deghan Manshadi, Lisa Ishiguro, Kyoung-Jin Sohn, Alan Medline, Richard Renlund, Ruth Croxford, Young-In Kim

**Affiliations:** 1 Department of Nutritional Sciences, University of Toronto and Keenan Research Center for Biomedical Science at St. Michael's Hospital, Toronto, Ontario, Canada; 2 Department of Medicine, University of Toronto and Keenan Research Center of Biomedical Science at St. Michael's Hospital, Toronto, Ontario, Canada; 3 Department of Laboratory Medicine & Pathobiology, University of Toronto, Toronto, Ontario, Canada; 4 Statistical Consultant, Toronto, Ontario, Canada; 5 Departments of Medicine & Nutritional Sciences, University of Toronto, Division of Gastroenterology, St. Michael's Hospital and Keenan Research Center of Biomedical Science at St. Michael's Hospital, Toronto, Ontario, Canada; 6 Department of Pathology, Humber River Regional Hospital, Toronto, Ontario, Canada; Columbia University, United States of America

## Abstract

Folic acid supplementation may prevent the development of cancer in normal tissues but may promote the progression of established (pre)neoplastic lesions. However, whether or not folic acid supplementation can promote the progression of established (pre)neoplastic mammary lesions is unknown. This is a critically important issue because breast cancer patients and survivors in North America are likely exposed to high levels of folic acid owing to folic acid fortification and widespread supplemental use after cancer diagnosis. We investigated whether folic acid supplementation can promote the progression of established mammary tumors. Female Sprague-Dawley rats were placed on a control diet and mammary tumors were initiated with 7,12-dimethylbenza[*a*]anthracene at puberty. When the sentinel tumor reached a predefined size, rats were randomized to receive a diet containing the control, 2.5x, 4x, or 5x supplemental levels of folic acid for up to 12 weeks. The sentinel mammary tumor growth was monitored weekly. At necropsy, the sentinel and all other mammary tumors were analyzed histologically. The effect of folic acid supplementation on the expression of proteins involved in proliferation, apoptosis, and mammary tumorigenesis was determined in representative sentinel adenocarcinomas. Although no clear dose-response relationship was observed, folic acid supplementation significantly promoted the progression of the sentinel mammary tumors and was associated with significantly higher sentinel mammary tumor weight and volume compared with the control diet. Furthermore, folic acid supplementation was associated with significantly higher weight and volume of all mammary tumors. The most significant and consistent mammary tumor-promoting effect was observed with the 2.5x supplemental level of folic acid. Folic acid supplementation was also associated with an increased expression of BAX, PARP, and HER2. Our data suggest that folic acid supplementation may promote the progression of established mammary tumors. The potential tumor-promoting effect of folic acid supplementation in breast cancer patients and survivors needs further clarification.

## Introduction

The role of folate, a water-soluble B vitamin, and its synthetic form, folic acid, in cancer development and progression is highly controversial [1], [2], [3]. Dietary intake and blood levels of folate appear to be inversely related to the risk of several malignancies, in particular colorectal cancer, in epidemiologic studies, although the strength, specificity and magnitude of this association have not been consistent [4]. For breast cancer, epidemiologic studies have not provided unequivocal support for an inverse association [5], [6], [7], [8]. However, folate status seems to interact with alcohol, a well-established risk factor for breast cancer and a folate antagonist, in modifying breast cancer risk; low folate intake increases, whereas high folate intake decreases, breast cancer risk among women who regularly consume moderate or high amounts of alcohol, but not among women with low or no alcohol consumption [6]. Recent epidemiologic studies have even suggested that high folate intake, largely from folic acid, [9], [10], [11], [12] and high plasma folate concentrations [13], [14] may increase breast cancer risk. The potential tumor-promoting effect of high folate status has been further suggested in other organs. The Aspirin/Folate Polyp Prevention Study reported that folic acid supplementation at 1 mg/day for 6–10 years significantly increased the risk of recurrence of advanced and multiple colorectal adenomas [15] and of prostate cancer [16] in predisposed subjects with prior colorectal adenomas who likely harboured preneoplastic foci. However, a combined analysis of 3 large randomized clinical trials of folic acid supplementation for the prevention of recurrent colorectal adenomas in those with an adenoma history including the Asprin/Folate Polyp Prevention study reported a null effect, although with shorter follow-up times [17]. Combined or meta-analyses of several randomized clinical trials that investigated the effect of folic acid supplementation with or without other B vitamins on cardiovascular disease outcomes as the primary endpoint reported either a tumor promoting [18] or null [19] effect on cancer risk as the secondary endpoint. Two most recent systemic review and meta-analysis of folic acid trials with cancer risk as either the primary or secondary endpoint again reported either an increased [20] or null [3], [21] effect associated with folic acid supplementation. With respect to breast cancer specifically, trials of folic acid, vitamin B6 and vitamin B12 conducted among individuals at high risk of cardiovascular disease found no effect on breast cancer outcomes [21], [22], [23], [24].

Animal studies conducted in colorectal cancer models have shown that folic acid supplementation prevents the development of cancer in normal tissues but promotes the progression of established (pre)neoplastic lesions [25], [26], [27], [28], [29]. Animal studies have also suggested that supraphysiologic supplemental doses of folic acid may promote, rather than prevent the cancer development [26]. Collectively, these observations suggest that folate possesses dual modulatory effects on cancer development and progression depending on the dose and the stage of cell transformation at the time of high folate exposure or folic acid supplementation [1], [2], [3], [4].

Folate intake and blood levels in North America have significantly increased over the past decade owing to mandatory folic acid fortification aimed at reducing the rate of neural tube defects and to widespread supplemental use of folic acid by up to 30–40% of the North American population [30], [31], [32]. Furthermore, vitamin and supplements are commonly used among patients newly diagnosed with cancer and cancer survivors with a much higher frequency of use compared with the general population (64–81% versus 50%) [33], [34], [35]. The highest prevalence of use is among Caucasian female breast cancer patients and survivors who are of middle to upper socioeconomic status [34]. A recent study of women with early-stage breast cancer has shown that 54% and 72% consume multivitamins pre- and post- cancer diagnosis, respectively [36], higher than the reported figure of 35% and 55% pre- and post-colorectal cancer diagnosis [35]. In another study, nearly 60% of African-American breast cancer survivors reported using multivitamin or folic acid supplement [37]. Among women at high risk of hereditary breast and ovarian cancer who underwent BRCA1/2 genetic testing for cancer susceptibility, 51% reported using at least one dietary supplement with 17% reporting multivitamin use [38].

Although the potential tumor-promoting effect of folic acid supplementation has been demonstrated for colorectal cancer in preclinical and human studies [1], [2], [4], whether or not folic acid supplementation can promote the progression of established (pre)neoplastic mammary lesions is unknown at present. This is a critically important issue because women with breast cancer or breast cancer survivors and women at high risk of developing breast cancer in North America are likely exposed to high levels of folate and folic acid in the post-fortification era. Given these considerations, we investigated whether folic acid supplementation can promote the progression of established mammary tumors in a well-established chemical carcinogen rodent model.

## Materials and Methods

### Animals, dietary intervention, and mammary tumor induction

**Figure 1:**
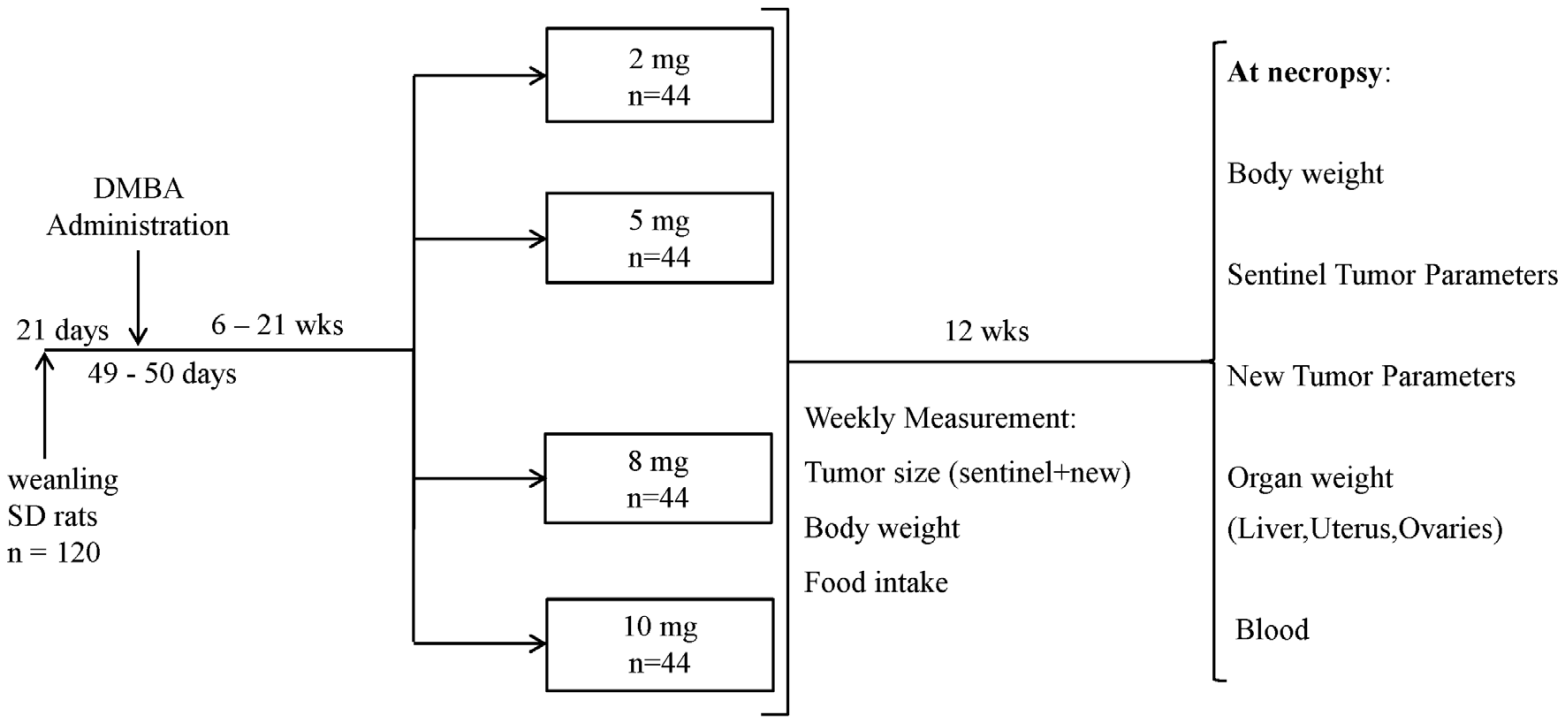
Study Design. Weanling female rats were placed on a control diet containing 2/kg diet (basal dietary requirement for rats) until 7 weeks of age when the mammary carcinogen DMBA was administered. The animals continued to receive the control diet until a single mammary tumor between 0.7–0.9 cm (i.e., the sentinel tumor) developed. The animals were then randomized to receive different levels of folic acid (control, 5, 8, and 10 mg folic acid/kg diet) for 12 weeks. At necropsy, all macroscopic mammary tumors (sentinel and new) were harvested and histologically confirmed as adenomas or adenocarcinomas. The primary objective of this study was to determine the effect of folic acid supplementation on the progression of the sentinel mammary tumors and on the sentinel tumor burden variables (weight, volume, and area). The secondary objective of this study was to determine the effect of folic acid supplementation on the incidence and tumor burden variables of all mammary tumors including the sentinel and new mammary tumors at necropsy.

This study was carried out in strict accordance with the Regulations of the Animals for Research Act in Ontario and the Guidelines of the Canadian Council on Animal Care. The protocol was approved by the Animal Care Committee of the University of Toronto (Permit Number: 20007988). Pathogen-free, 3-week-old female Sprague-Dawley rats (∼50 g; Charles River Laboratories, St. Constant, Quebec, Canada) were placed on an amino acid-defined diet (Dyets, Bethlehem, PA) containing 2 mg (control) folic acid/kg diet (Figure 1). At puberty (7 weeks of age), all animals received a single intragastric dose of 5 mg of 7,12-dimethylbenza[*a*]anthracene (DMBA; Sigma-Aldrich, St. Louis, MO) dissolved in 1.0 mL of corn oil for mammary tumor induction [39], [40] and continued to receive the control diet until a single mammary tumor between 0.7–0.9 cm (i.e., the sentinel tumor) developed. The animals were then randomized to receive the same amino acid-defined diet containing 2 (control), 5, 8 or 10 mg folic acid/kg (n = 44 per dietary group) for 12 weeks (Figure 1).

Amino acid-defined diets containing different levels of folic acid constitute a standard method of providing supplemental dietary folic acid in rodents and have been extensively used in previous studies of dietary folate and cancer in rodents [25], [26], [27], [28], [29], [41] including mammary tumors [42], [43], [44]. The control diet containing 2 mg of folic acid/kg diet is generally accepted as the basal dietary requirement (BDR) for rats [45]. This diet contains approximately 4000 kcal/kg diet, which translates to 0.5–1 mg of folic acid in 2000 kcal. This level of folic acid expressed relative to caloric content very closely approximates the recommended daily allowance (RDA) of 0.4 mg dietary folate equivalent in humans consuming a daily average of 2000 kcal [46] and thus, was selected to parallel the RDA for folate in humans. The diet containing 5 mg folic acid/kg diet (2.5x BDR) was selected to approximate the likely total folate intake (∼0.8–1.0 mg folic acid/day or 2–2.5x RDA) from fortified foods and multivitamin containing 0.4 mg folic acid in North American populations in the postfortification era [1]. Also, this selected supplemental level of folic acid approximates the recommended dose of folic acid supplementation (up to 1.0 mg/day or 2.5x RDA) for all women planning a pregnancy or capable of becoming pregnant [47]. The 8 mg and 10 mg folic acid/kg diet (4x and 5x BDR, respectively) were selected to approximate ∼1.6–2 mg folic acid/day (4–5x RDA) in humans, reflecting the levels that might be consumed by a subgroup of the North American population who receive ≥1 mg folic acid/day for certain medical conditions in the postfortification era [1]. The detailed composition of the diets has been published previously [29]. Diets and water were provided *ad libitum* prior to randomization while post-randomization the total food intake in each dietary group was matched to that of the group with the lowest food intake.

### Observational parameters

Body weights and food intake were recorded weekly. All rats were palpated for mammary tumors once a week beginning at 4 weeks post-DMBA administration. The number, size, and location of each tumor were recorded in a manner such that, after histological diagnosis, the primary and secondary outcomes could be limited to adenomas and adenocarcinomas. Sentinel tumor progression per week was estimated using length^2^×width^2^×0.5×1/body weight and corrected for age at randomization and days on diet. All rats were monitored daily for clinical evidence of illness or morbidity and those approaching a predefined moribund state [42], [43] were promptly killed.

### Sample collection and analysis of mammary tumors

Rats were killed by carbon dioxide inhalation followed by cervical dislocation. At necropsy, blood was collected and plasma was stored at −80°C with 0.5% ascorbic acid for plasma folate determination and without ascorbic acid for plasma homocysteine assay. The liver, ovaries, and uterus were removed and weighed rapidly. The liver and normal mammary tissues from each rat were excised, snap-frozen, and stored at −80°C for determination of hepatic and mammary folate concentrations.

All macroscopic mammary tumors were counted, excised, and weighed, and all three diameters from each tumor were measured using a digital calliper for computation of final tumor volume (length×width×[height×½]). All macroscopic mammary tumors were fixed and processed in a standard manner for H&E staining and independently analyzed histologically by 2 study pathologists (R.R. and A.M.) who were blinded to the study group.

### Folate and homocysteine concentrations

Plasma and tissue folate concentrations were determined by a standard microbiological microtiter plate assay [42], [43]. Total plasma homocysteine concentrations were determined using the Axis Homocysteine EIA kit (Abbott Laboratories, Mississauga, Ontario, Canada) [27].

### Western blot analysis

Six representative sentinel adenocarcinomas from each diet group were selected for protein expression analysis. The selected sentinel adenocarcinomas within each dietary group had the final tumor weight and volume and plasma folate concentrations close to the mean of their respective group. Furthermore, these selected tumors were not significantly different with respect to the age at randomization, days on diet, final animal weight, and tumor area at the time of randomization. The expression of several proteins involved in cell proliferation, apoptosis, and breast carcinogenesis was determined by standard Western blot analysis as described previously [48] using a rabbit polycolonal antibody at a dilution of 1∶1000 for PARP (Cell Signaling; catalogue #9542), BAX (catalogue # 2772), BCL-XL (catalogue # 2762), BCL-2 (catalogue # 2876), caspase-3 (catalogue # 9662), and HER2 (catalogue # 3250) and at a 1∶500 dilution for ERα (Santa Cruz; catalogue # sc-543) and using a mouse monoclonal antibody at a dilution of 1∶2000 dilution for PCNA (Cell Signaling; catalogue #2586). To confirm that the proteins were loaded equally, the membranes were stripped and re-probed with a mouse monoclonal antibody at a dilution of 1∶3000 for β-actin (Sigma, Oakville, ON). Densitometry of bands were determined using the public domain ImageJ (version 1.38) from the National Institute of Health available on the Internet at http://rsbweb.nih.gov/ij. The protein expression is reported as % protein/β actin.

### Statistical analysis

Continuous variables were log-transformed due to departure from normality. All main primary and secondary outcomes used general linear regression. Analyses of characteristics at the time of randomization were adjusted for age at randomization. Analyses of the main primary and secondary outcomes incorporated one factor (diet) and two covariates (age at randomization and days on diet). Age at randomization and days on diet were entered as covariates to correct for their potential confounding effects. Binary logistic regression was used to analyze the distribution of adenocarcinomas and adenomas within each diet group. Body weight, food intake, and sentinel tumor rate of progression were analyzed using repeated measures analyses. Correlations between continuous dependent variables were evaluated using the Spearman's rank correlation test. The expression of the selected proteins was analyzed using one-way ANOVA followed by a Tukey post hoc test for group comparisons. All significance tests were two-sided and were considered significant at p<0.05. Results are expressed as mean ± SEM unless stated otherwise. Statistical analyses were performed using SPSS 19.0 for Windows (SPSS, Chicago, IL).

## Results

### Body weight, daily food consumption, and clinical characteristics

At randomization the animals in the four treatment groups did not differ significantly in terms of mean body weight, mean sentinel tumor area, or mean age (Table 1). During the first week post-randomization, the animals on the 2 and 10 mg folic acid/kg diets had a higher initial weight gain compared with those on the 8 mg folic acid/kg diet (p<0.05) whereas the initial weight gain of those on the 5 mg folic acid/kg diet did not differ significantly from other diet groups. This difference in initial weight gain during the first week post-randomization disappeared by the second week of dietary intervention. The mean daily food consumption, which was determined on a pre-assigned day of each week, was not significantly different among the four dietary groups during the study period.

**Table 1:** Characteristics at randomization and at necropsy.

	Diet group	2	5	8	10	p-value
**At randomization**	**Body weight (g)**	360.9±8.6^a^	351.6±11.7	358.5±6.5	356.9±4.3	0.90
		353.5 (259.0–523.0)^b^	350.0 (282.0–465.0)	354.0 (291.0–454.0)	354.4 (270.0–471.0)	
	**Sentinel tumor area (mm^2^)**	59.8±6.9	60.6±4.8	51.9±3.3	58.2±4.0	0.59
		45.4 (26.5–239.2)	52.0 (27.8–208.3)	45.4 (24.5–128.0)	53.2 (26.5–189.7)	
	**Age (days)**	134.4±3.8	134.2±4.0	136.3±4.1	135.8±3.8	0.98
		130 (94–196)	130 (100–196)	127 (99–196)	130 (92–196)	
**At necropsy**	**Days on diet**	63.1±2.4	58.1±1.7	56.5±1.6	59.4±1.7	0.80
		84 (8–86)	77 (13–86)	64 (22–86)	77 (11–86)	

Diet groups represent the amount of folic acid in mg per kg diet.

a Results in the first row of each category are expressed as mean ± SEM.

b Results in the second row of each category are median values (range of minimum and maximum values).

No animals died prematurely, nor was any animal euthanized before the scheduled necropsy (i.e., 12 weeks post-dietary randomization) due to clinical evidence of illness, morbidity, or moribund state. However, >50% of animals from each dietary group had to be euthanized before the scheduled necropsy due to large and/or ulcerated tumors. The proportion of animals euthanized before the scheduled necropsy was not significant different among the four dietary groups (range 50–61%). As a result, the mean duration of dietary intervention ranged from 56.5 to 63.1 days across the four dietary groups but was not significantly different among the four dietary groups at necropsy (Table 1).

The mean organ weights of the liver, ovaries, and uterus were not significantly different among the four dietary groups. Furthermore, the mean weights of these organs after correcting for body weight, age at randomization, and days on diet were not significantly different among the four dietary groups.

### Systematic and tissue folate status

At necropsy, the mean plasma folate concentrations were significantly different among the four dietary groups (p = 0.0001; Table 2), increasing with increased dietary folic acid levels (Table 2). The mean mammary gland folate concentrations of the three folic acid supplemented dietary groups were not significantly different from each other but were significantly higher than that of the control group (p = 0.0001; Table 2) and increased with increasing dietary folate. Similarly, mean hepatic folate concentrations increased incrementally as the dietary levels of folic acid increased (p = 0.013); the animals on the 10 mg folic acid/kg diet had significantly higher hepatic folate concentrations than those receiving the 2 mg folic acid/kg diet and the animals on the 5 and 8 mg folic acid/kg diet had the values intermediate between the 2 and 10 mg folic acid/kg diet groups (Table 2). Hepatic folate concentrations were significantly correlated with plasma folate concentrations (r = 0.20; p = 0.009). The mean plasma concentrations of homocysteine (an accurate inverse functional indicator of folate status [49]) of the animals receiving the 8 and 10 mg folic acid/kg diets were significantly lower than that of those on the 2 mg folic acid/kg diet (p = 0.005; Table 2) whereas the value of the animals on the 5 mg folic acid/kg diet was intermediate between the control and two higher supplemental levels of folic acid. Plasma homocysteine concentrations were inversely and significantly correlated with plasma folate concentrations (r = −0.26; p = 0.0001).

**Table 2:** Folate and homocysteine concentrations at necropsy.

Diet group	2	5	8	10	p-value
**Plasma folate (ng/mL)**	60.8±1.0^a^	86.7±1.0^b^	102. 3±1.0^c^	119.4±1.0^d^	0.0001
**Mammary folate (ng/g tissue)**	59.8±1.1^a^	77.8±1.1^b^	86.9±1.1^b^	94.2±1.1^b^	0.0001
**Hepatic folate (µg/g tissue)**	6.5±0.5^a^	7.0±0.5^a,b^	7.3±0.5^a,b^	8.5±0.5^b^	0.013
**Plasma homocysteine (µmol/L)**	7.0±0.2^a^	6.5±0.2^a,b^	6.1±0.2^b^	6.0±0.2^b^	0.005

Diet groups represent the amount of folic acid in mg per kg diet. Results are expressed as mean ± SEM. Within each row, means with different letters significantly differ at p<0.05. All analyses were adjusted for the effects of age at randomization and days on diet.

### Effect of folic acid supplementation on sentinel mammary tumor progression and tumor burden variables

At necropsy, sentinel mammary tumors were histologically confirmed as adenocarcinomas or adenomas. The proportion of sentinel adenocarcinomas was not significantly different among the four dietary groups (73–88%; p = NS; Table 3). The primary objective of this study was to determine the effect of folic acid supplementation on the progression of sentinel mammary tumors during the dietary intervention and on the sentinel tumor burden variables (weight, volume, and area) at necropsy. We considered adenomas and adenocarcinomas together as sentinel tumors given the well-established mammary adenoma to adenocarcinoma progression in the DMBA rat model [39], [40].

**Table 3:** Summary of the effects of folic acid supplementation on main primary outcomes of the sentinel mammary tumors.

Diet group	2	5	8	10	p-value
**Sentinel adenocarcinoma incidence (%)**	72.5	87.8	85.0	76.7	NS
**Sentinel adenoma incidence (%)**	27.5	12.2	15.0	23.3	NS
**Sentinel tumor weight (g)**	0.7±1.2^a^	1.6±1.2^b^	1.3±1.2^b^	1.2±1.2^b^	0.001
**Sentinel tumor volume (cm^3^)**	0.6±1.2^a^	1.4±1.2^b^	1.2±1.2^b^	1.1±1.2^b^	0.001
**Sentinel tumor area (mm^2^)**	146.2±1.1^a^	235.0±1.1^b^	215.7±1.1^b^	208.4±1.1^a,b^	0.007

Weanling female rats were placed on a control diet containing 2 mg kg folic acid/kg diet (basal dietary requirement for rats) until 7 weeks of age when the mammary carcinogen DMBA was administered. The animals continued to receive the control diet until a single mammary tumor between 0.7–0.9 cm (i.e., the sentinel tumor) developed. The animals were then randomized to receive different levels of folic acid (control, 5, 8, and 10 mg folic acid/kg diet) for 12 weeks. The primary objective of this study was to determine the effect of folic acid supplementation on the progression of the sentinel mammary tumors including both histologically confirmed adenocarcinomas and adenomas and on the sentinel tumor burden variables (weight, volume, and area). Diet groups represent the amount of folic acid in mg per kg diet. Results are expressed as mean ± SEM. Within each row, means with different letters significantly differ at p<0.05. All analyses were adjusted for the effects of age at randomization and days on diet.

**Figure 2:**
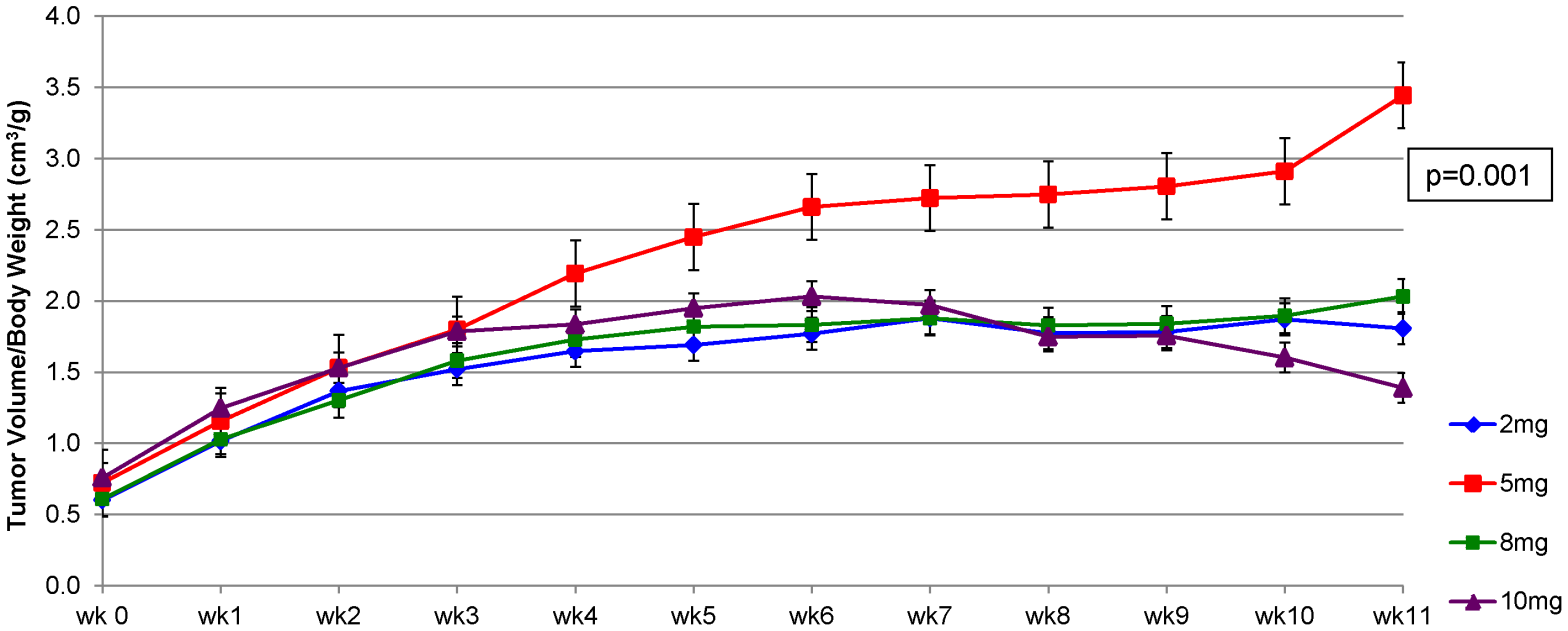
Effect of folic acid supplementation on the progression of sentinel mammary tumors. Folic acid supplementation at 5/kg diet significantly accelerated the progression of sentinel mammary tumor over time compared with the control diet and two higher supplemental levels of folic acid after correcting for age at randomization and days on diet (p = 0.001).

Folic acid supplementation at 5 mg/kg diet accelerated the progression of sentinel mammary tumor volume over time compared with the control diet and two higher supplemental levels of folic acid after correcting for age at randomization and days on diet (p = 0.001; Figure 2). However, significant acceleration of sentinel mammary tumor progression was observed only during the later stage of dietary intervention (Figure 2). No significant difference in the rate of sentinel tumor progression was observed among the 2, 8, and 10 mg folic acid/kg diet groups. A similar pattern of sentinel mammary tumor progression was observed when only sentinel adenocarcinomas were considered, although the accelerated sentinel adenocarcinoma progression in the 5 mg folic acid group was no longer statistically significant (p = 0.12).

At necropsy, sentinel mammary tumors in animals receiving the three supplemental levels of folic acid were associated with significantly higher final sentinel tumor weight and volume (p = 0.001) and significantly larger final sentinel tumor area compared with those in animal receiving the control diet after correcting for age at randomization and days on diet (p = 0.007; Table 3). However, no significant differences in these sentinel mammary tumor characteristics among the three supplemental levels of folic acid, nor was there a significant dose-response relationship. Similar to the sentinel tumor progression data, sentinel mammary tumors in animals receiving the 5 mg folic acid/kg diet had the highest final sentinel tumor weight and volume and the largest final sentinel tumor area, although these observations did not reach statistical significance. When only sentinel mammary adenocarcinomas were considered, the three supplemental levels folic acid were associated with significantly higher sentinel adenocarcinoma weight and volume (p<0.04), but not area, compared with the control diet, again without a significant dose-response relationship. No significant correlations were observed between plasma folate, mammary folate, or plasma homocysteine concentrations and sentinel mammary tumor weight, volume, or area.

Tumor latency (time to appearance of the sentinel mammary tumor) ranged from 5–22 weeks post-DMBA administration in the present study. Although we have corrected for this potential confounder in the analysis, we performed a subanalysis considering animals randomized between 8 and 15 weeks post-DMBA administration. In order to capture 65% of the entire sample, we considered animals whose age at randomization was ±1 SD of the median (129 days) (104–154 days). Furthermore, the duration for which the animals were on the diet ranged from 7 days to 85 days. Although we have corrected for this potential confounder in the analysis, we performed a subanalysis considering animals that were on the diet for a minimum of 30 days. The results from both subanalyses were similar to those when all the animals were included in the analysis (data not shown).

### Effect of folic acid supplementation on all mammary tumor (sentinel+new) outcomes

We also examined the effect of folic acid supplementation on all mammary tumors including the sentinel and new mammary tumors. Consistent with previous observations [39], [40], [42], [43], [44], >80% of macroscopic mammary lesions were identified histologically as adenomas (19%; range 12.2–21.4%) or adenocarcinomas (81%; range 78.6–87.8%). The proportion of mammary adenocarcinomas and adenomas was not significantly different among the four dietary groups (Table 4).

**Table 4:** Summary of the effects of folic acid supplementation on all (sentinel+new) mammary tumors.

Diet group	2	5	8	10	p-value
**Adenocarcinoma incidence (%)**	78.6	87.8	81.8	83.2	NS
**Adenoma incidence (%)**	21.4	12.2	18.2	16.8	NS
**Mean of all tumor weight (g)**	2.0±1.2^a^	3.7±1.2^b^	3.2±1.2^a,b^	2.9±1.2^a,b^	0.03
**Mean of all tumor volume (cm^3^)**	1.8±1.2^a^	3.3±1.2^b^	2.6±1.2^a,b^	2.5±1.2^a,b^	0.04
**Mean of all tumor burden (mm)**	48.2±1.1	57.2±1.1	58.5±1.1	51.8±1.1	NS
**Mean tumor multiplicity**	3.4±0.4	4.5±0.4	4.4±0.4	3.7±0.4	NS

The secondary objective of this study was to determine the effect of folic acid supplementation on the incidence and tumor burden variables of all mammary tumors including the sentinel and new mammary adenomas and adenocarcinomas at necropsy. Diet groups represent the amount of folic acid in mg per kg diet. Results are expressed as mean ± SEM. Within each row, means with different letters significantly differ at p<0.05. All analyses were adjusted for the effects of age at randomization and days on diet.

Folic acid supplementation was associated with higher mean weight (p = 0.03) and volume (p = 0.04) of all mammary tumors compared with the control diet (Table 4) after correcting for age at randomization and days on diet; however, a statistically significant difference was observed only between the 5 mg folic/kg diet and control group; the values of the 8 and 10 mg folic acid/kg diet groups were intermediate between the control and 5 mg folic acid/kg diet groups (Table 4). Folic acid supplementation was associated a nonsignificant trend toward increased tumor multiplicity (the mean number of mammary tumors per animal) (p = 0.09; Table 4) but folic acid supplementation had no significant effect on mammary tumor burden (the sum of all mammary tumor diameters per animal) (Table 4). No significant correlation was observed between plasma folate, mammary folate, or plasma homocysteine concentrations and total mammary tumor weight, volume, burden, or multiplicity (p = NS). Similar observations were made when only mammary adenocarcinomas were considered in the analysis (data not shown).

Two subanalyses considering animals whose age at randomization was between 104–154 days of age and animals that were on the diet for a minimum of 30 days were performed. Again, the results from both subanalyses were similar to those when all the animals were included in the analysis (data not shown).

### Effect of folic acid supplementation on the expression of proteins involved in proliferation, apoptosis and mammary carcinogenesis

**Figure 3:**
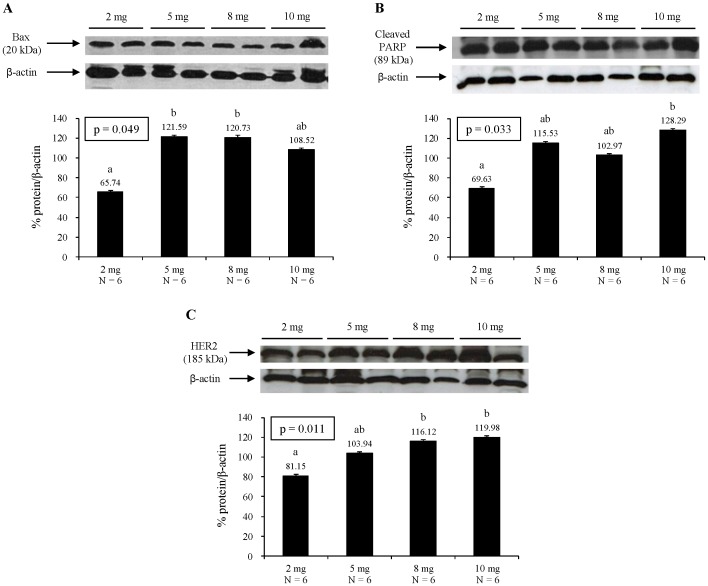
Effect of folic acid supplementation on Bax (A), PARP (B), and HER2 (C) protein expression. Representative Western blot is presented on top and densitometric quantification of protein expression is presented as % protein/β actin at the bottom of each panel. Values are mean ± SEM. Different letters denote significant differences at p<0.05. All analyses were corrected for the effects of age at randomization and days on diet.

We also examined the effect of folic acid supplementation on proliferation (PCNA), apoptosis (PARP, caspase-3, BAX, BCL-2, and BCL-XL), and mammary carcinogenesis (ERα and HER2) by determining the expression of several proteins involved in these processes in carefully selected representative sentinel adenocarcinomas from each diet group. Folic acid supplementation had no significant effect on the expression of PCNA (proliferating cell nuclear antigen), a co-factor for DNA polymerase delta that helps increase the processivity of leading strand synthesis during DNA replication [50] (data not shown). Folic acid supplementation had no significant effect on the expression of the anti-apoptotic proteins BCL-2 and BCL-XL or caspase-3, a member of the cysteine-aspartic acid protease. Sequential activation of caspase-3 plays a central role in the execution-phase of apoptosis [51] (data not shown). However, folic acid supplementation at 5 and 8 mg/kg diet was associated with significantly higher expression of the pro-apoptotic protein BAX compared with the control diet (p<0.05) while folic acid supplementation at 10 mg/kg diet had a protein expression value intermediate between the control and the 5 and 8 mg folic/kg diet groups (Figure 3A). Folic acid supplementation incrementally increased the expression of PARP (poly [ADP-ribose] polymerase) (p = 0.033; Figure 3B). PARP is a DNA-repair enzyme that can deplete cellular ATP stores when it catalyzes the repair of multiple DNA strand breaks that occur in cell injury [52]. In apoptosis, PARP undergoes rapid cleavage and inactivation (detection of cleaved PARP is a diagnostic test for apoptosis), so stores of ATP are preserved [52]. ATP is necessary for numerous effector processes in apoptosis, whereas exhaustion of ATP shifts the cell from apoptosis to necrosis [52]. Folic acid supplementation also incrementally increased the expression of HER2 (human epidermal growth factor receptor 2) (p = 0.011; Figure 3C), a member of the epidermal growth factor receptor family. Amplification or over-expression of HER2 has been shown to play an important role in the pathogenesis and progression of breast cancer [53]. Folic acid supplementation had no significant effect on the expression of ERα (data not shown).

## Discussion

In the present study, we specifically determined the potential tumor-promoting effect of folic acid supplementation on established mammary tumors in the well-established DMBA rat model of mammary tumorigenesis. Based on the biological function of folate and the previously observed tumor-promoting effect of folic acid supplementation made in preclinical colorectal cancer models, our *a priori* hypothesis was that folic acid supplementation would promote the progression of established mammary tumors. Whether or not folic acid supplementation would promote the progression of breast cancer is a significant public health concern. Cancer patients and survivors in North America have a high prevalence of multivitamin and supplement use with breast cancer patients and survivors having the highest prevalence [33], [34], [35], [36], [37], [38]. Furthermore, folate intake and blood levels in North America have significantly increased over the last 15 years owing to mandatory folic acid fortification and widespread supplemental use of folic acid by up to 30–40% of the North American population [30], [31], [32]. Therefore, women with breast cancer or breast cancer survivors in North America are likely exposed to high levels of folate and folic acid in the post-fortification era. Although no clear dose-response relationship was observed, our data indeed demonstrated that folic acid supplementation between 2.5x and 5x the BDR significantly promoted the progression of the sentinel mammary tumors and was associated with significantly higher sentinel mammary tumor weight, volume, and area at necropsy compared with the control diet. Interestingly, the most significant and consistent mammary tumor-promoting effect was observed with the lowest supplemental level of folic acid (2.5x the BDR). Furthermore, folic acid supplementation was associated with significantly higher weight and volume of all mammary tumors including the sentinel and new mammary tumors with folic acid supplementation at 2.5x the BDR demonstrating the most significant and consistent effect.

Although the tumor-promoting effect of folic acid supplementation has been demonstrated for colorectal cancer in preclinical and human studies [1], [2], [4], this is the first study to demonstrate the potential tumor-promoting effect of folic acid supplementation on mammary tumors. One important distinction is that the animal studies using colorectal cancer models have investigated the effect of folic acid supplementation on the progression of pre-neoplastic lesions of colorectal cancer (e.g., aberrant crypt foci) [25], [26], [27], [28], [29] whereas our study examined the effect of folic acid supplementation on the progression of established mammary tumors. Previous animal studies using the well-established N-methyl-N-nitrosourea (MNU) rat model have demonstrated that neither a mild degree of dietary folate deficiency nor folic acid supplementation (4–20x the BDR) significantly modulates the initiation phase of the MNU-induced mammary tumorigenesis [42], [43], [54]. In contrast, folate deficiency inhibited, whereas folic acid supplementation did not significantly affect, the promotion phase of the MNU-induced mammary tumorigenesis [42], [43], [54]. These observations made in the MNU rat model of mammary tumorigenesis suggest that folic acid supplementation may not have a significant effect at the promotion and/or early progression phase of mammary tumorigenesis. In these studies that investigated the effect on the promotion phase, folic acid supplementation was provided one week following MNU administration at puberty (50 days of age). In contrast, in the present study, we started folic acid supplementation at an average of 12 weeks following DMBA administration when the sentinel mammary tumor reached a predefined size post-DMBA administration (19 weeks of age). In this setting, folic acid supplementation significantly promoted the progression of DMBA-induced mammary tumors in the progression phase. It would be of great interest to investigate the effect of folic acid supplementation provided during the promotion and/or early progression phases of mammary tumorigenesis in the DMBA rat model.

We carefully considered and deliberately selected the supplemental levels of folic acid in the present study. The diet containing 5 mg folic acid/kg diet (2.5x BDR) was selected to approximate the likely total folate intake (∼0.8–1.0 mg folic acid/day or 2–2.5x RDA) from fortified foods and multivitamin containing 0.4 mg folic acid in North American populations in the postfortification era [1]. The 8 mg and 10 mg folic acid/kg diet (4x and 5x BDR, respectively) were selected to approximate ∼1.6–2 mg folic acid/day (4–5x RDA) in humans, reflecting the levels that might be consumed by a subgroup of the North American population who receive ≥1 mg folic acid/day for certain medical conditions in the postfortification era [1]. However, because of inherent differences in folate metabolism between human and rats [55], [56], our selected dietary folic acid levels may not accurately reflect the corresponding levels in humans. Folic acid is not found in nature nor is it a normal metabolite. It must be reduced, first to dihydrofolate and then to tetrahydrofolate by dihydrofolate reductase and methylated to 5-methyltetrahydrofolate (the predominant folate found in blood), in the liver and to a lesser degree in the intestine, before it can enter the folate cycle [56]. Recent evidence suggests that rats have a comparatively high dihydrofolate reductase activity relative to humans [55], [56]. Consequently, rats would have to ingest a much greater than pro-rata amount of folic acid in order to elicit the same circulating plasma concentrations of folic acid as humans in order to assess the impact of systemic exposure of folic acid [55], [56]. Therefore, the selected supplemental levels of dietary folic acid in rats in the present study likely achieved much lower plasma concentrations of folic acid than would have been achieved by the equivalent supplemental levels of folic acid in humans. Nevertheless, the selected modest supplemental levels of folic acid significantly enhanced the progression of established mammary tumors. It would be interesting to determine whether higher supplemental levels of folic acid demonstrate a greater magnitude of and more consistent and unequivocal evidence for the tumor-promoting effect of folic acid supplementation.

Mechanistically, the most probable pathway by which folic acid supplementation increases the progression of established mammary tumors is the provision of nucleotide precursors to rapidly replicating neoplastic cells for accelerated proliferation and uncontrolled cell growth [1], [4]. Folate mediates the transfer of one-carbon units involved in *de novo* purine and thymidylate synthesis and hence is an essential cofactor for DNA synthesis and replication [57]. We examined the expression of several proteins involved in proliferation (PCNA), apoptosis (PARP, caspase-3, BAX, BCL-2, and BCL-XL and mammary carcinogenesis (ERα and HER2) in carefully selected representative sentinel adenocarcinomas from each diet group. Interestingly, folic acid supplementation had no significant effect on the expression of PCNA, the anti-apoptotic proteins BCL-2 and BCL-XL, or caspase-3. However, folic acid supplementation appeared to promote apoptosis in the sentinel mammary tumors as evidenced by increased expression of the pro-apoptotic protein BAX as well as PARP. However, it is known that during the early stages of tumor expansion, growth is exponential, but with enlargement, tumor growth slows at the late stages [58]. Therefore, the late stages of tumor growth might be less proliferative and more likely to undergo apoptosis. Unless determined in normal mammary tissues or in early preneoplatic stages, markers of proliferation and apoptosis in established mammary tumors may not appropriately reflect the effect of folic acid supplementation on proliferation and apoptosis. One interesting observation is that folic acid supplementation incrementally increased the expression of HER2, a member of the epidermal growth factor receptor family, amplification or over-expression of which has been shown to play an important role in the pathogenesis and progression of breast cancer [53]. This observation needs to be confirmed and further studies are warranted to elucidate possible mechanisms by which folic acid supplementation may upregulate HER2. In this regard, a recent study has suggested a possible link between frequent promoter cytosine-guanine dinucleotide (CpG) island DNA methylation of 11 genes and HER2 amplification in human breast cancers [59]. Folate is involved in remethylation of homocysteine to synthesize methionine, which is a precursor of S-adenosylmethionine, the primary methyl group donor for most biological methylation reactions [60]. In this role, therefore, folate plays an important role in DNA methylation, an epigenetic determinant in gene expression (an inverse relation except for few exceptions), in the maintenance of DNA integrity and stability, in chromatin modifications, and in the development mutations [60]. Therefore, one possible mechanism by which folic acid supplementation may upregulate HER2 may be CpG DNA hypermethylation.

We did not observe a clear dose-dependent effect of folic acid supplementation on mammary tumor progression in the present study. Although the most significant and consistent tumor promoting effect was observed with 5 mg folic acid supplementation, essentially, no significant difference was observed among the three supplemental levels of folic acid. This may be related to the fact that mammary gland folate concentrations seemed to reach a plateau beyond the 5 mg folic acid/kg diet and were not significantly different among the three supplemental levels of folic acid. This finding is probably due to the fact that folate accumulation in tissues is limited by the level of folylpolylglumate synthase activity in the setting of substrate excess [57]. Furthermore, high circulating and tissue levels of unmetabolized folic acid has been shown to inhibit dihyrofolate reductase (DHFR) [55], the primary enzyme that reduces and methylates folic acid, thereby decreasing the intracellular folate pool and consequently inhibiting cellular proliferation [61], [62], [63], [64]. Also, DHFR inhibition resulting from high folic acid can downregulate thymidylate synthase, the key enzyme in DNA synthesis and replication, because the transcription of these genes is co-regulated by several several transcritpon factors [65] and this would result in decreased DNA synthesis. These observations, therefore, collectively suggest that the effect of folic acid supplementation on cell proliferation and growth is likely non-linear and reaches a plateau at a certain threshold. Several mathematical models of folate cycle and metabolism have also reported this non-linear relationship [66], [67]. Because we wished to determine the effect of folic acid supplementation on established mammary tumors, we did not initiate dietary intervention until the sentinel mammary tumor reached a diameter between 0.7–0.9 cm. As a result, a wide variation in tumor latency (time to appearance of the sentinel mammary tumor), the age of the animal at randomization, and the number of days the animals were on the diet was observed. We corrected for these potential confounding factors and also performed subanalyses, which did not change the results. An alternative approach is to start dietary intervention at a fixed time point after DMBA administration in all animals regardless of the appearance of a sentinel mammary tumor; this approach is for studies aimed at examining the effect of folic acid supplementation on the promotion or early progression phase of mammary tumorigenesis.

DMBA-induced mammary tumorigenesis in rats is different from that in human breast cancer in several important aspects: (i) the use of the genotoxic chemical carcinogen and (ii) molecular genetic differences (lack of *p53* and *BRCA* mutations) [39], [40]. Nonetheless, the DMBA rat model is widely used to determine the effects of dietary factors on mammary tumorigenesis for the following reasons: (i) histological similarities of adenocarcinoma to human breast cancer; (ii) molecular genetic similarities (*Erb2/HER2*, *TGFβ*, and *cyclinD1*); (iii) a clear operational distinction between initiation and promotion stages; (iv) hormonally dependent mammary tumorigenesis; and (vi) expression of estrogen and progesterone receptors [39], [40].

In summary, notwithstanding the limitations associated with the DMBA rat model of mammary tumorigenesis and inherent differences in folate metabolism between humans and rats, our data suggest, for the first time, that folic acid supplementation between 2.5x and 5x the basal dietary requirement significantly promotes the progression of established mammary tumors. These supplemental levels are easily achievable in the North American population in the post folic acid fortification era [30], [31], [32] and in particular breast cancer patients and survivors who tend to have the highest levels of supplement use post cancer diagnosis [33], [34], [35], [36], [37], [38]. Although we did not interrogate comprehensive mechanisms in the present study, our data suggest that the tumor-promoting effect of folic acid supplementation may be related to an upregulation of HER2. Given the fact that women with breast cancer or breast cancer survivors in North America are likely exposed to high levels of folate and folic acid in the post-fortification era, the question of whether or not folic acid supplementation promotes the progression of breast cancer needs further clarification. Definitive answers about the potential tumor-promoting effect of folic acid supplementation on (pre)neoplastic lesions in the mammary glands are beyond the reach of both observational epidemiologic and intervention trials in humans. However, our data from the present animal study, in conjunction with biologically plausible mechanisms relating to folate's biochemical function in one-carbon transfer reactions and with the tumor-promoting effect of folic acid supplementation on (pre)neoplastic colorectal lesions observed in preclinical and clinical studies, suggest that there is sufficient cause for concern about the potentially deleterious effect of folic acid supplementation on breast cancer progression.
